# Case report: Bitter vertigo

**DOI:** 10.3389/fneur.2022.1028597

**Published:** 2022-10-06

**Authors:** Nicolina Goldschagg, Christian Brem, Michael Strupp

**Affiliations:** ^1^Department of Neurology and German Center for Vertigo and Balance Disorders, Ludwig Maximilians University, Munich, Germany; ^2^Institute of Neuroradiology, University Hospital, Ludwig Maximilians University, Munich, Germany

**Keywords:** vertigo, basilar artery aneurysm, vestibular paroxysmia, case report, dysgeusia, dolichoectasia

## Abstract

**Background:**

There are many causes of episodes of vertigo and very few causes of episodes of changes in taste, and the combination of the two is very rare. Here, we describe a patient with recurrent short episodes of vertigo in combination with simultaneous episodes of recurrent paroxysmal dysgeusia and altered feeling on the left side of face. The symptoms were caused by compression of the vestibulocochlear nerve and the facial nerve due to dolichoectasia of the basilar artery.

**Methods:**

The patient was diagnosed in our routine clinical practice and underwent a complete neurological and neuro-otological examination, including video head impulse test, caloric irrigation, ocular and cervical vestibular evoked myogenic potentials, acoustic-evoked potentials, neuro-orthoptic examination, cranial MRI, and MR angiography. The patient was seen twice for follow-up.

**Case:**

A 71-year-old patient primarily presented with a 2-year history of recurrent short episodes of spinning vertigo. Each of the episodes began with an altered feeling on the left side of the face, followed by a bitter taste on the left half of the tongue, and subsequently vertigo lasting for up to 15 s. The frequency of the attacks was high: up to 80 times per day. Laboratory tests revealed signs of a peripheral vestibular deficit on the left side. There were no signs of sensory or motor deficits or of altered taste between the episodes. An MRI of the brain showed an elongated basilar artery leading to an indentation of the facial and vestibulocochlear nerves on the left side.

**Conclusion:**

We propose a neurovascular compression in the proximal part of two cranial nerves because of pulsatile compression by the elongated basilar artery with ephatic discharges as the cause of the recurrent episodes. Consistent with the theory of ephatic discharges, treatment with the sodium channel blocker lacosamide for over six months with a final dosage of 200 mg per day p.o. led to a significant reduction of the attack frequency and intensity. This treatment option with a sodium channel blocker should therefore not only be considered in vestibular paroxysmia but also in cases of paroxysmal dysgeusia.

## Introduction

There are a several common causes of changes in taste, including chemotherapy or infections, but paroxysmal dysgeusia has rarely been described (1). This case report describes a combination of vestibular, sensory, and gustatory symptoms due to compression of two cranial nerves because of dolichoectasia of the basilar artery. Recurrent short oligosymptomatic episodes of vertigo are also rare and are the leading symptom of vestibular paroxysmia (2), most often caused by neurovascular compression.

## Case description

A 71-year-old patient presented with a 2-year history of recurrent very short episodes of spinning vertigo. Each of the episodes started with an altered feeling on the left side of the face, followed by a bitter taste on the left half of the tongue, and subsequently vertigo lasting for up to 10 to 15 s. He had a high frequency of attacks of up to 80 times per day.

The patient was seen in our routine clinical practice in the neurological outpatient clinic of LMU Munich. He underwent a complete neurological, neuro-otological, and neuro-ophthalmological examination, including video head impulse test, caloric irrigation, ocular- and vestibular-evoked myogenic potentials, acoustic-evoked potentials, neuro-orthoptic examination, cranial MRI, and MR angiography. The patient was seen twice for follow-up: after a treatment period of 6 months and after 4 years without medication.

## Diagnostic assessment

The neurological examination revealed horizontal head-shaking nystagmus to the right. There were no signs of persisting sensory or motor deficits or of a taste deficit between the attacks. The video head impulse test showed a decreased gain of the VOR (gain on the left: 0.54, right: 0.83) and the caloric testing reduced excitability on the left side (mean peak slow phase velocity on the left with cold water 6.1°/s, with warm water 4.7°/s, on the right with cold water 13.2°/s, and with warm water 21.3°/s; side difference 52%). The amplitudes of the ocular vestibular-evoked myogenic potentials were reduced on the left side (p1-n1 amplitude: left 3.7μV, right 5.4 μV), as were the amplitudes of the cervical vestibular-evoked myogenic potentials (p1-n1 amplitude: left 74 μV, right 102 μV) (for details please refer to Supplementary Figure 1). The acoustic-evoked potentials were normal.

**Figure 1:**
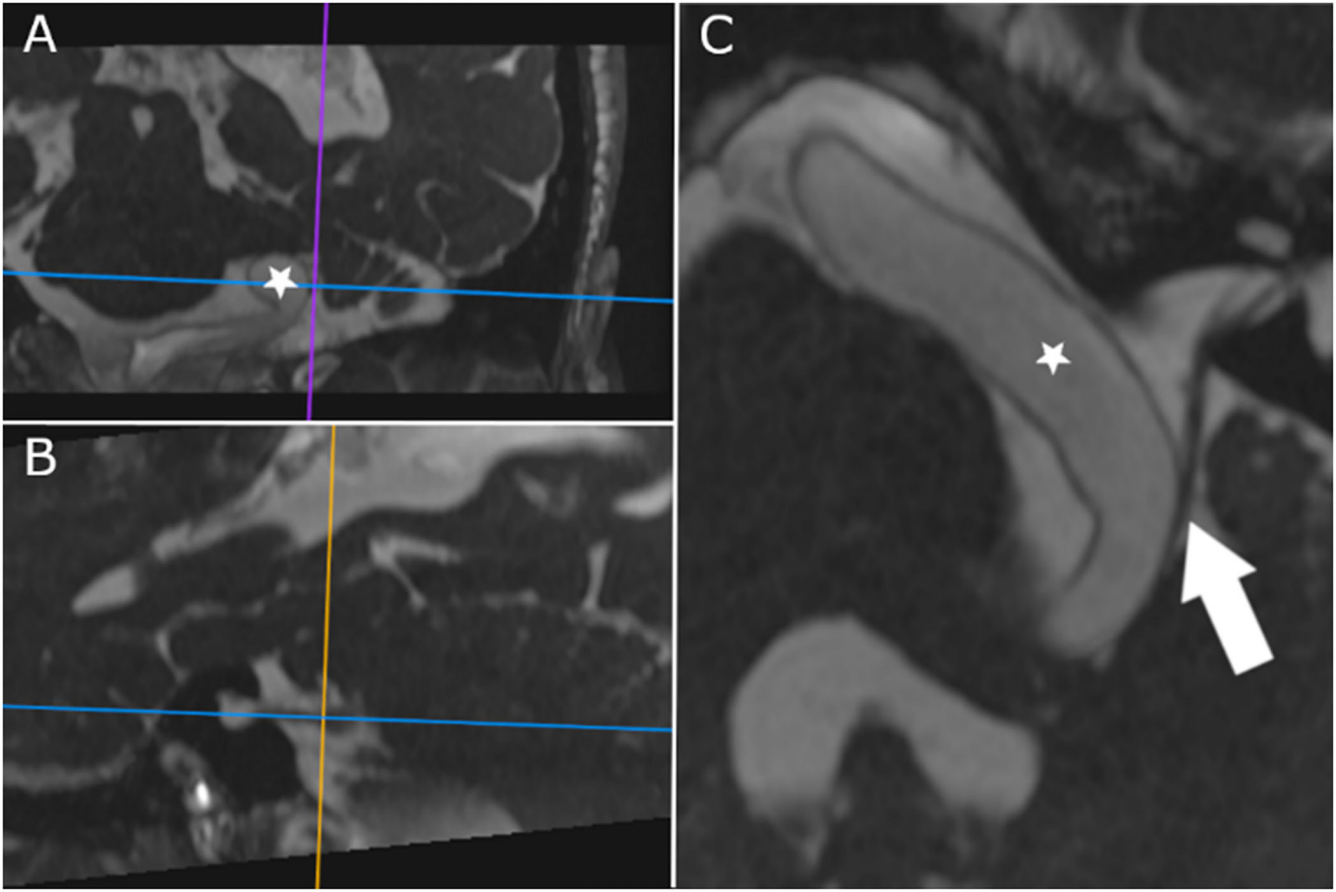
CISS-MRI in oblique axis reconstructed along the cisternal course of the vestibulocochlear and facial nerves (the horizontal line in **(A,B)** depicts the axis of **(C)**; vertical lines delineate the axis of the other planes, **(A,B)**, respectively; lines are centered on contact point of the vestibulocochlear nerve and the basilar artery). The dilated and elongated basilar artery (star) displaces the nerves, and direct contact is shown (arrow).

The MRI of the brain showed an elongated basilar artery leading to an indentation of the facial and vestibulocochlear nerves on the left side (Figure 1). On the MRI, no contact between the elongated basilar artery and the trigeminal and glossopharyngeal nerves was seen. There was also no evidence of brainstem edema, and no macroangiopathy or stenosis of the neck vessels was detectable on ultrasound.

The treatment with initially lacosamide 50 mg in the evening and later 200 mg per day p.o. led to a significant and stable reduction in the attack intensity and frequency from 80 to 1–2 attacks per day over nearly 5 months. The medication was stopped after the follow-up visit after 6 months at the request of the patient.

The follow-up visit after 4 years revealed 5–10 attacks per day without medication. The video head impulse test during the follow-up after 4 years still showed a decreased gain of the VOR (left: 0.71, right: 1.09) and caloric testing progressive reduced excitability on the left side (mean peak slow phase velocity on the left with cold water 2.8°/s, with warm water 2.3°/s, on the right with cold water 20.8°/s, and with warm water 9.2°/s; side difference 71%). The amplitudes of the ocular vestibular-evoked myogenic potentials were low on both sides (p1-n1 amplitude: left 1.1 μV, right 1.7 μV), and the cervical vestibular-evoked myogenic potentials were reduced on the left side (p1-n1 amplitude: left 70 μV, right 187 μV) (for details please refer to Supplementary Figure 2).

## Discussion/Conclusion

Basilar artery dolichoectasia has a prevalence of about 0.8 to 4% (3), with higher values in older men, as in this case. Intracranial arterial dolichoectasia is well-known in patients with stroke, with an incidence of up to 17% in patients with posterior circulation stroke and over 45 years of age (4). A dolichoectatic artery can also lead to compression of cranial nerves with well documented case reports of hemifacial spasm (5), trigeminal neuralgia (6) or vestibular paroxysmia (7) or a combination of several symptoms. For instance, there are patients who have, in addition to recurrent episodes of vertigo, other neurological symptoms and signs such as facial hemispasm (8, 9) due to combined irritation of cranial nerves VIII and VII in the meatus acusticus internus where both nerves lie close to each other. There is also a description of the combination of trigeminal neuralgia and facial hemispasm (10).

Here, we describe an evidently very rare triad of unilateral facial sensory, gustatory, and vestibular symptoms due to compression of two cranial nerves: the facial and the vestibulocochlear nerves.

First, the unilaterally impaired sensation in the face is well-known in patients with prior isolated facial nerve dysfunction, as in Bell's palsy (11, 12); this is probably due to impairment of visceral efferent fibers of the facial nerve. Another possible explanation for the sensory symptoms in facial nerve palsy is impaired processing of the facial somatosensory information (11).

Second, the gustatory symptoms can be explained by an affection of the visceral afferent fibers of the facial nerve. These fibers rise from the ipsilateral nucleus tractus solitarius and reach the anterior two-thirds of the tongue via the chorda tympani and the lingual nerve. In this case, this leads to altered and bitter taste. Since these visceral fibers are un-myelinated and motor fibers are myelinated, this may explain why the patient had no facial hemispasm.

Third, the vestibular symptoms are similar to those of vestibular paroxysmia.

In our patient, the MRI showed no brainstem edema or compression of other cranial nerves as alternative explanations for the patient's symptoms.

Because of the short episodes with a duration of less than 1 min and a stereotypical phenomenology, we propose the ephatic discharges as the cause for the above-described symptoms, similar to other neurovascular compression syndromes of cranial nerves. Pulsatile compression of the nerves by an elongated basilar artery leads to pathological ephatic transmission between neighboring axons of the affected nerves.

Sodium channel blockers, and especially carbamazepine, are well-known treatment options for cranial neuralgias and may stabilize the cell membrane (13). For vestibular paroxysmia, a placebo-controlled trial showed a benefit of oxcarbazepine (14). Lacosamide is a third-generation antiepileptic drug and selectively enhances slow inactivation of voltage-gated sodium channels. Studies on patients with epilepsy showed that lacosamide is well-tolerated (15) and has a better tolerability profile than carbamazepine (16), leading to less withdrawal due to side effects. Consistent with the theory on ephatic discharges, treatment with the sodium-channel blocker lacosamide led to a significant reduction of the attack frequency and intensity in our patient. Lacosamide is therefore a well-tolerated treatment option for patients with cranial nerve compression syndromes, especially vestibular paroxysmia (17), trigeminal neuralgia, or their combination, as shown in this case.

## Take-away lesson from the case

We present a patient with a unique triad of recurrent short episodes of vestibular, unilateral facial sensory, and gustatory symptoms due to compression of the facial and vestibulocochlear nerves by an elongated basilar artery. Consistent with the theory on ephatic discharges, treatment with lacosamide 200 mg per day p.o. led to a significant reduction of the attack frequency and intensity in our patient. Based on this case report, sodium channel blockers can also be a new treatment option for patients with paroxysmal dysgeusia.

## Data availability statement

The raw data supporting the conclusions of this article will be made available by the authors, without undue reservation.

## Ethics statement

Ethical review and approval was not required for the study on human participants in accordance with the local legislation and institutional requirements. The patients/participants provided their written informed consent to participate in this study. Written informed consent was obtained from the individual(s) for the publication of any potentially identifiable images or data included in this article.

## Author contributions

NG: analysis and interpretation of the data and drafting/revision of the manuscript. CB: analysis and interpretation of the data, figure design, and critical revision of the manuscript for intellectual content. MS: examination, diagnosis and follow-up of the patient, concept, analysis and interpretation of the data, and critical revision of the manuscript for intellectual content. All authors contributed to the article and approved the submitted version.

## Conflict of interest

Author MS is Joint Chief Editor of the Journal of Neurology, Editor in Chief of Frontiers of Neuro-otology, and Section Editor of F1000. He has received speaker's honoraria from Abbott, Auris Medical, Biogen, Eisai, Grünenthal, GSK, Henning Pharma, Interacoustics, J&J, MSD, NeuroUpdate, Otometrics, Pierre-Fabre, TEVA, UCB, and Viatris. He receives support for clinical studies from Decibel, USA, Cure within Reach, USA and Heel, Germany. He distributes “M-glasses” and “Positional vertigo App”. He acts as a consultant for Abbott, Auris Medical, Heel, IntraBio, Sensorion, and Vertify. The remaining authors declare that the research was conducted in the absence of any commercial or financial relationships that could be construed as a potential conflict of interest.

## Publisher's note

All claims expressed in this article are solely those of the authors and do not necessarily represent those of their affiliated organizations, or those of the publisher, the editors and the reviewers. Any product that may be evaluated in this article, or claim that may be made by its manufacturer, is not guaranteed or endorsed by the publisher.

## Supplementary material

Supplementary Figure 1 Examination results, first visit in 2018. **(A)** Caloric testing showed reduced excitability on the left side (mean peak slow phase velocity on the left with cold water 6.1°/s, with warm water 4.7°/s, on the right with cold water 13.2°/s, and with warm water 21.3°/s; side difference 52%). **(B)** Video head impulse test showed decreased gain of the VOR (gain on the left: 0.54, right: 0.83) **(C)** Amplitudes of the ocular vestibular-evoked myogenic potentials were reduced on the left side (p1-n1 amplitude: left 3.7 μV, right 5.4 μV). **(D)** Amplitudes of the cervical vestibular-evoked myogenic potentials were reduced on the left side (p1-n1 amplitude: left 74 μV, right 102 μV).

Supplementary Figure 2 Examination results, follow-up in 2022. **(A)** Caloric testing during the follow-up after 4 years showed progressive reduced excitability on the left side (mean peak slow phase velocity on the left with cold water 2.8°/s, with warm water 2.3°/s, on the right with cold water 20.8°/s, and with warm water 9.2°/s; side difference 71%). **(B)** Video head impulse test during the follow-up after 4 years still showed a decreased gain of the VOR (left:0.71, right: 1.09). **(C)** Amplitudes of the ocular vestibular-evoked myogenic potentials were low on both sides (p1-n1 amplitude: left 1.1 μV, right 1.7 μV). **(D)** Amplitudes of the cervical vestibular-evoked myogenic potentials were reduced on the left side (p1-n1 amplitude: left 70 μV, right 187 μV).
