# Supplementary figures and images for: Case report: Bitter vertigo
Source: Front Neurol. 2022 Oct 6;13:1028597. doi: 10.3389/fneur.2022.1028597 (PMC9582759; doi:10.3389/fneur.2022.1028597)

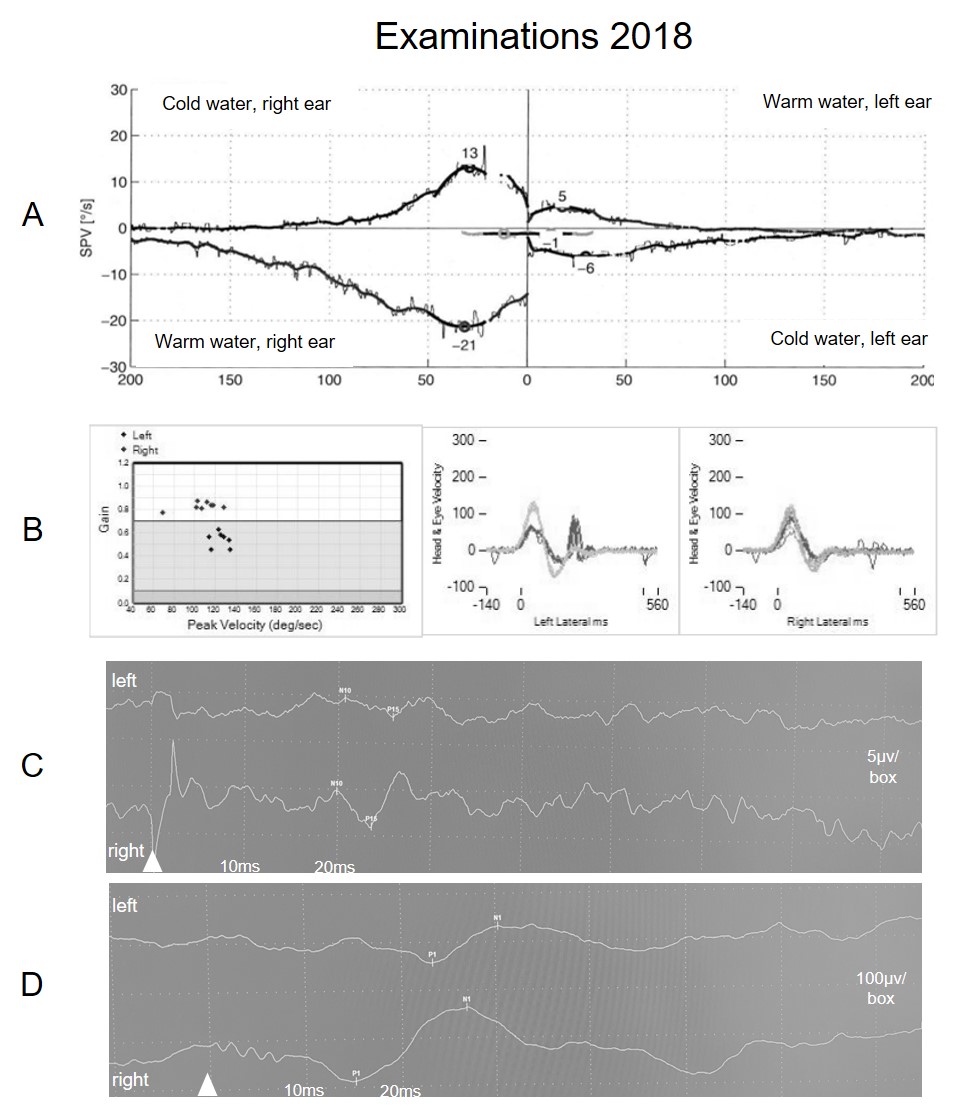

Supplement: Supplementary Figure 1 — Examination results, first visit in 2018. (A) Caloric testing showed reduced excitability on the left side (mean peak slow phase velocity on the left with cold water 6.1°/s, with warm water 4.7°/s, on the right with cold water 13.2°/s, and with warm water 21.3°/s; side difference 52%). (B) Video head impulse test showed decreased gain of the VOR (gain on the left: 0.54, right: 0.83) (C) Amplitudes of the ocular vestibular-evoked myogenic potentials were reduced on the left side (p1-n1 amplitude: left 3.7 μV, right 5.4 μV). (D) Amplitudes of the cervical vestibular-evoked myogenic potentials were reduced on the left side (p1-n1 amplitude: left 74 μV, right 102 μV). [file Image_1.jpg]

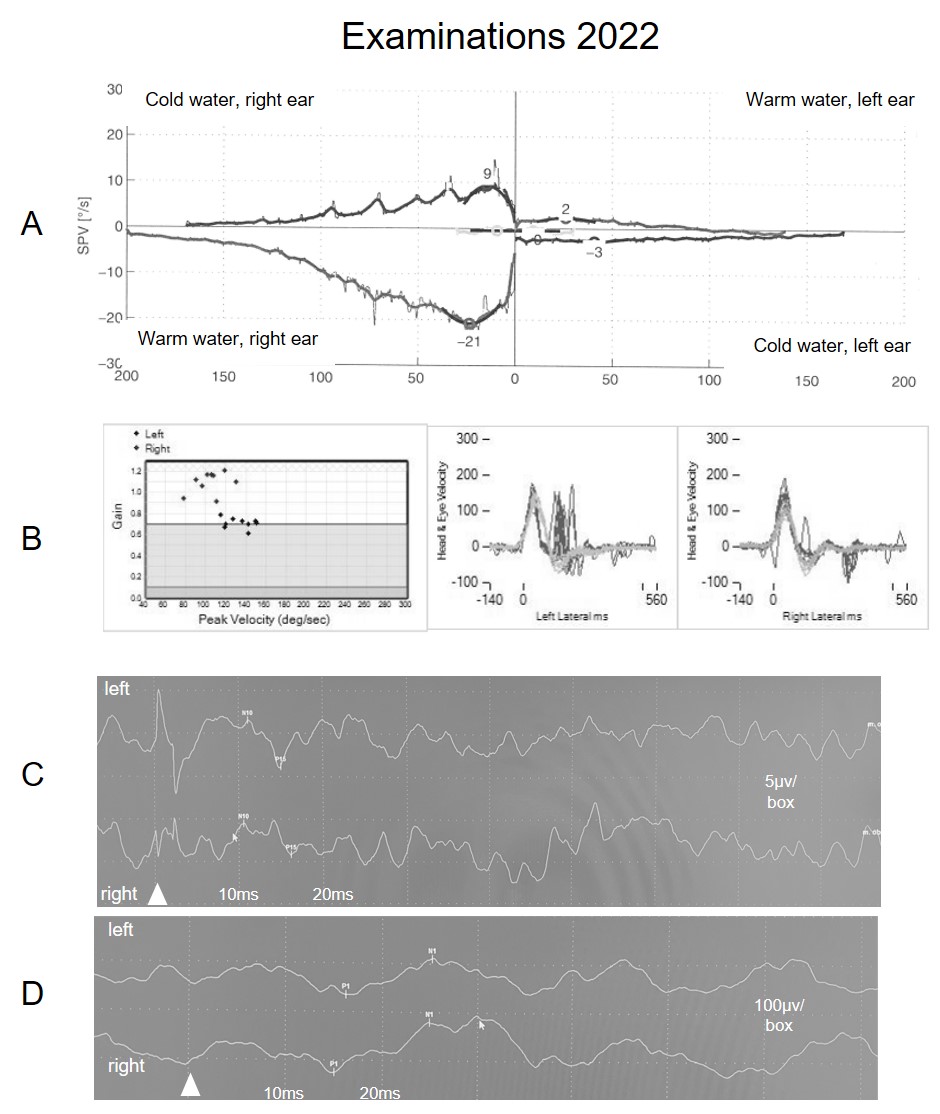

Supplement: Supplementary Figure 2 — Examination results, follow-up in 2022. (A) Caloric testing during the follow-up after 4 years showed progressive reduced excitability on the left side (mean peak slow phase velocity on the left with cold water 2.8°/s, with warm water 2.3°/s, on the right with cold water 20.8°/s, and with warm water 9.2°/s; side difference 71%). (B) Video head impulse test during the follow-up after 4 years still showed a decreased gain of the VOR (left:0.71, right: 1.09). (C) Amplitudes of the ocular vestibular-evoked myogenic potentials were low on both sides (p1-n1 amplitude: left 1.1 μV, right 1.7 μV). (D) Amplitudes of the cervical vestibular-evoked myogenic potentials were reduced on the left side (p1-n1 amplitude: left 70 μV, right 187 μV). [file Image_2.jpg]
